# Prevalence of depressive symptoms among Italian medical students: The multicentre cross-sectional “PRIMES” study

**DOI:** 10.1371/journal.pone.0231845

**Published:** 2020-04-17

**Authors:** Fabrizio Bert, Giuseppina Lo Moro, Alessio Corradi, Anna Acampora, Antonella Agodi, Laura Brunelli, Maria Chironna, Silvia Cocchio, Vincenza Cofini, Marcello Mario D’Errico, Carolina Marzuillo, Cesira Pasquarella, Maria Pavia, Vincenzo Restivo, Maria Rosaria Gualano, Paolo Leombruni, Roberta Siliquini, Collaborating Group

**Affiliations:** 1 Department of Public Health Sciences, University of Torino, Torino, Italy; 2 A.O.U. City of Health and Science of Torino, Torino, Italy; 3 Sezione di Igiene, Istituto di Sanità Pubblica, Università Cattolica del Sacro Cuore, Roma, Italia; 4 Department of Medical and Surgical Sciences and Advanced Technologies "GF Ingrassia", University of Catania, Catania, Italy; 5 Department of Medicine, University of Udine, Udine, Italy; 6 Department of Biomedical Sciences and Human Oncology, University of Bari—Aldo Moro, Bari, Italy; 7 Department of Cardiac, Thoracic, Vascular Sciences and Public Health, University of Padova, Padova, Italy; 8 Biostatistics and Epidemiology Unit, Department of Life, Health and Environmental Sciences, University of L'Aquila, L'Aquila, Italy; 9 Department of Biomedical Sciences and Public Health, Polytechnic University of Marche, Ancona, Italy; 10 Department of Public Health and Infectious Diseases, Sapienza University of Roma, Roma, Italy; 11 Department of Medicine and Surgery, University of Parma, Parma, Italy; 12 Department of Health Sciences, University "Magna Graecia" of Catanzaro, Catanzaro, Italy; 13 Department of Science for Health Promotion and Mother to Child Care "G. D'Alessandro", University of Palermo, Palermo, Italy; 14 Clinical Psychology and Psycho-Oncology Unit, Department of Neurosciences, University of Torino, Torino, Italy; Uniformed Services University of the Health Sciences, UNITED STATES

## Abstract

**Background:**

Four percent of the world’s population suffers from depression, which is a major public health issue. Medical students are at risk, as their depressive symptoms (DS) prevalence is reported to be approximately 27% worldwide. Since few data on Italian medical students exist, this study aimed to estimate their DS prevalence and assess risk and protective factors.

**Methods:**

The PRIMES was a multicentre cross-sectional study performed in 12 Italian medical schools. Questionnaires were self-reported and included 30 sociodemographic items and the Beck Depression Inventory-II (BDI-II). The primary outcome was the presence of DS (BDI-II score≥14). The main analyses were chi-squared tests and multivariable logistic regressions with a p-value<0.05 considered significant.

**Results:**

The number of collected questionnaires was 2,513 (117 BDI-II incomplete). Females accounted for 61.3% of the respondents, and the median age was 22 years (IQR = 4). The prevalence of DS was 29.5%. Specifically, 14.0% had mild depression, 11.1% had moderate depression, and 4.5% had severe depression. The main risk factors for DS were age, being female, bisexual/asexual orientation, living with partner/housemates, poor economic status (worsened by living far from home), less than 90 min of weekly exercise, relatives with psychiatric disorders, personal chronic disease, judging medical school choice negatively, unsatisfying friendships with classmates, competitive and hostile climate among classmates, thinking that medical school hinders specific activities and being worried about not measuring up to the profession. Protective factors included family cohesion, hobbies, intellectual curiosity as a career motivation and no worries about the future.

**Conclusion:**

Italian medical students are at high risk of reporting DS, similar to the global population of medical students’. Medical schools must make efforts to implement preventive and treatment interventions by offering counselling and working on modifiable factors, such as lifestyle and learning climate.

## Introduction

Depressive disorders represent a major public health issue, as 4% of the world’s population suffers from these conditions; depressive disorders are also the single largest contributor to non-fatal health loss worldwide, causing 5% of all Years Lived with Disability, with an increasing trend [1]. Depressive disorders consist of two groups, both presenting depressive symptoms (DS): major depressive disorder/depressive episode and dysthymia [2].

Recently, attention on the relationships between DS and medical students has grown, as medical students have been recognized as a high-risk population [3]. An overview concluded that the pooled DS prevalence among medical students was 27.0% (95% confidence interval (CI), 24.7–29.5%) [3]. The European prevalence was 20.1% (95% CI, 15.8–25.1%); however, no meta-analyses included Italian studies [3]. This situation seems to persist in residents and is associated with low-quality care and augmented errors, in addition to the burden of depression per se [4]. Unfortunately, medical students seek help infrequently; only 15.7% of depressed students undergo treatments [5]. Among the main reasons, the avoidance of appropriate help-seeking behaviour is related to viewing mental problems as a weakness and an obstacle to one’s career [6].

Given the above, mental health services for medical students are expanding, and a growing body of work is studying interventions [7,8]. Medical schools have the duty to help with supportive and preventive strategies. To organize effective plans, it is important to have information on the steps to become a medical doctor (MD) and collect data on the mental conditions of students. In Italy, each medical school has a fixed number of places for the students, assigned through a yearly admission test. The test is nationwide: students might need to move to attend medical school. The school lasts 6 years: the first three years focus on biological subjects, and the last three focus on clinical subjects, with rotations in the hospital that usually begin at the 4^th^ year. The grading system uses a 30-point scale, with 18 as the threshold for passing exams. The grade average is one of the means to obtain bonus points for admission tests to specialty schools, which also have a fixed number of places, yearly admission tests and nationwide rankings. Admission is highly competitive for medical schools and for specialty schools; for example, in 2018, approximately 1/7 and 1/2 of applicants were admitted to medical schools and specialty schools, respectively [9].

Since no data on Italian medical students were reported in the most relevant works [3], the present study aimed to estimate the DS prevalence among these students and to assess risk and protective factors, making a comparison to factors identified in other medical student populations [5,10]. In particular, this project aimed to explore how DS is affected by some of the abovementioned key components of the Italian medical school context, such as the possibility of moving away from home, the need to keep a good grade average and the worry about the competition for specialty school admission. The final aim is to thoroughly examine all these aspects to have a basis for implementing preventive interventions.

## Methods

The Psychosocial Report in Italian MEdical Students (PRIMES) was a multicentre cross-sectional study conducted in 12 medical schools (29.27% of Italian medical schools [11]). Schools were selected by convenience to represent the whole country: 33.3% were located in Northern Italy, 25% in Central Italy and 41.7% in Southern Italy (ISTAT classification [12]). Raosoft^®^ was used to determine that the minimum sample size was 383, based on a 5% margin of error, 95% confidence level, 50% response distribution and population of 78,101 (medical students in 2017 [11]).

All procedures performed were in accordance with the 1964 Helsinki declaration and its later amendments. The Ethics Committee of the University of Torino approved the protocol. Participants were asked to sign an informed consent form and were recruited by opportunity sampling in the 1^st^, 4^th^ and 6^th^ years of their programmes in participating schools to assess DS at the beginning, in the middle and at the end of their academic path (approximately 9,000 eligible students). The enrolment occurred far from exams (November 2018). Participation was voluntary and anonymous, and participants received no compensation.

### The questionnaire

The questionnaire was self-administered. A 30-item sociodemographic part was developed after a literature review on depression-related factors in medical students, and the Beck Depression Inventory-II (BDI-II) [13] was used to assess DS.

The independent variables were coded from the sociodemographic items, which were designed to test hypotheses found in the literature. The sociodemographic items assessed the following possible risk factors: sexual orientation [14]; family history of psychiatric disorders and suicide [15,16]; personal chronic disease [15,17]; distance from home [6,10]; poor economic status [15,17]; and working while in medical school due to financial need [17,18]. Distance from home and economic status were recoded as one variable to examine if their interaction could worsen DS. The following possible protective factors were investigated: social support, e.g., living condition and family cohesion [17,19]; relationship status [20]; exercise [21]; and hobbies/extracurricular activities [22]. It has been reported that depressive risk is higher when students perceive that university does not leave time for other activities [23]; therefore, the survey asked if medical school impeded exercise or other activities. Other medical school-related information was gathered because career motivations and opinions about the choice of medical school might influence mental health [24]. Consequently, judgements about medical school choice and motivations behind it were assessed. Since the academic curriculum has been reported to affect mental health, grade average, grade satisfaction and being on time with exams were investigated in the 4^th^ and 6^th^ years [10,25]. A grade average ≥27/30 was considered high. Climate among classmates and satisfaction with friendships with classmates were assessed because stress/anxiety produced by competitiveness and low social support could lead to depression [5,26]. Since fears about the medical profession could influence mental health [6,10], worries about the future were assessed.

Finally, items about stimulants [27], psychiatric medications and psychologist/psychiatrist follow-up were assessed to evaluate potential under-treatments reported as a risk in medical students [5]. Similarly, the survey asked if participants knew about university psychological counselling and if they would use it in case of need.

The presence of DS was assessed using the BDI-II, a 21-item self-report instrument that measures the severity of DS over the last two weeks. Each item has a score of 0 to 3, and the total score is the sum of all items. Total scores from 0–13 represent no/minimal depression, 14–19 represent mild depression, 20–28 represent moderate depression, and 29–63 represent severe depression [13]. The threshold of 14 has been used in several studies to examine the prevalence of DS, as reported recently in a meta-analysis and in an overview of systematic reviews [3,5]. Therefore, we decided to dichotomize our outcome by referring to a score ≥14 to represent DS presence. Moreover, this tool can be separated into affective, cognitive and somatic subscales (details in Table 4) [28]. Since there are no subscale scoring categories, these variables were analysed as continuous.

### Statistical analysis

Descriptive analyses were performed for all variables (categorical variables expressed as frequencies and percentages). Age, BDI-II total score and subscale score were expressed as the median and interquartile range (IQR) since the Shapiro-Wilk test indicated that these data did had non-normal distributions. DS prevalence was calculated with a 95% bootstrap CI (simple random sampling method). Chi-squared tests and adjusted residuals were computed to assess differences between years, far/not far from home participants, and students with/without DS (Kruskal-Wallis and Mann-Whitney U test for continuous variables). Additionally, Chi-squared tests among students with/without DS were stratified by year.

The effects of the independent variables on DS were analysed with multivariable logistic regressions adjusted for age and gender, both examining the whole sample and stratifying by year (details in Table 3). Final models were achieved with a backward stepwise method (results expressed as odds ratios OR, 95% CI).

To assess whether independent variables were differently associated with subscales, multivariable linear regressions adjusted for age and gender were performed for each subscale (details in Table 4). Outlier removal was based on Cook’s distance, and variables were selected by backward elimination (results expressed as unstandardized coefficients B, 95% CI).

SPSS software (version 25) was used, and a two-tailed p-value<0.05 was considered to be statistically significant. Missing values were excluded.

## Results

### Characteristics of the sample

A total of 2,513 questionnaires were collected (28% of eligible participants). The descriptive analyses were performed on all questionnaires, while the analyses about DS were performed on 2,396 questionnaires (117 BDI-II scales were incomplete).

Females accounted for 61.3% of the population, and the median age was 22 (IQR = 4). Participants studying far from home accounted for 60.4% of the population. Psychiatric medication consumption during university occurred in 5.7% of students, and 6.1% of students were seeing a psychologist/psychiatrist; 6^th^-year students reported higher frequencies for both variables (p<0.001). A total of 38.9% of respondents knew about their university counselling, with 1^st^-year students less likely to know (p<0.001). A total of 42.7% of participants reported that they would use university counselling; 1^st^-year students were more prone to use counselling, while 4^th^-year students were less prone (p = 0.008). Other data are presented in Table 1.

**Table 1:** Overall characteristics of the whole sample and grouped by year of course attended: Descriptive and chi-squared analysis (2018).

Characteristic		Overall	1^st^ year	4^th^ year	6^th^ year	p*
		(n = 2513)	(n = 1041)	(n = 744)	(n = 728)	
		N (%)	N (%)	N (%)	N (%)	
Age**		22 (4)	19 (1)	22 (1)	24 (1)	**<0.001**
**Gender**						
*Male*		969 (38.7)	410 (39.5)	283 (38.2)	276 (38.0)	0.778
*Female*		1536 (61.3)	628 (60.5)	458 (61.8)	450 (62.0)	
**Medical school geographical area**						
*Northern Italy*		995 (39.6)	416 (40.0)	295 (39.7)	284 (39.0)	0.096
*Central Italy*		563 (22.4)	244 (23.4)	178 (23.9)	141 (19.4)	
*Southern Italy*		955 (38.0)	381 (36.6)	271 (36.4)	303 (41.6)	
**Nationality**						
*Italian*		2468 (98.6)	1012(97.7)^b^	735 (99.1)	721 (99.3)^a^	**0.007**
*Other*		36 (1.4)	24 (2.3)^a^	7 (0.9)	5 (0.7)^b^	
**Relationship status**						
*Single*		1218 (48.6)	576 (55.6)^a^	355 (47.8)	287 (39.5)^b^	**<0.001**
*Involved*		1286 (51.4)	460 (44.4)^b^	387 (52.2)	439 (60.5)^a^	
**Sexual orientation**						
*Heterosexual*		2144 (86.1)	896 (87.2)	632 (85.5)	616 (85.3)	0.096
*Homosexual*		45 (1.8)	11 (1.1)	19 (2.6)	15 (2.1)	
*Bisexual*		283 (11.4)	116 (11.3)	85 (11.5)	82 (11.4)	
*Asexual*		17 (0.7)	5 (0.5)	3 (0.4)	9 (1.2)	
**Living condition**						
*Alone*		119 (4.7)	49 (4.7)	33 (4.4)	37 (5.1)	0.087
*With parents/relatives*		1261 (50.3)	552 (53.1)	347 (46.7)	362 (49.9)	
*With partner/housemates*		1129 (45.0)	439 (42.2)	363 (48.9)	327 (45.0)	
**Family cohesion**						
*Very poor/poor/excessive*		277 (11.1)	99 (9.5)^b^	85 (11.5)	93 (12.8)	**<0.001**
*Good*		935 (37.3)	345 (33.3)^b^	306 (41.2)^a^	284 (39.1)	
*Excellent*		1293 (51.6)	593 (57.2)^a^	351 (47.3)^b^	349 (48.1)^b^	
**Working because of income need**						
*No job/job not for income need*		2400 (95.7)	1007 (96.8)	707 (95.0)	686 (94.8)	0.061
*Yes*		108 (4.3)	33 (3.2)	37 (5.0)	38 (5.2)	
**Distance from home and economic status**						
*Not far from home*:	*Good status*	873 (35.2)	370 (36.2)	235 (31.8)	268 (31.8)	**0,043**
	*Poor status*	107 (4.3)	39 (3.8)	31 (4.2)^b^	37 (5.1)	
*Far from home*:	*Good status*	1354 (54.6)	552 (54.0)	416 (56.4)	386 (53.5)	
	*Poor status*	148 (6.0)	62 (6.1)	56 (7.6)^a^	30 (4.2)^b^	
**Having at least one hobby**^#^		1741 (69.8)	772 (74.7)^a^	483 (65.4)^b^	486 (67.2)	**<0.001**
**Exercising less than 90 min weekly**^#^		1435 (57.2)	631 (60.8)^a^	388 (52.2)^b^	416 (57.3)	**0.001**
**1**^**st**^**/2**^**nd**^ **degree relatives with psychiatric disorders**^#^		595 (23.8)	209 (20.1)^b^	180 (24.3)	206 (28.5)^a^	**<0.001**
**Suicides/suicide attempts** ^#^**among 1**^**st**^**/2**^**nd**^ **degree relatives**^#^		147 (5.9)	58 (5.6)	43 (5.8)	46 (6.3)	0.797
**Having a chronic disease**^#^		180 (7.2)	37 (3.6)^b^	47 (6.3)	96 (13.2)^a^	**<0.001**
**Taking stimulants to improve academic performance**^#^		125 (5.0)	39 (3.8)^b^	53 (7.1)	33 (4.5)^a^	**0.005**
**Judging the choice of medical school**						
*Positively*		1983 (79.4)	906 (87.5)^a^	572 (77.1)	505 (70.0)^b^	**<0.001**
*Negatively/No opinion*		516 (20.6)	130 (12.5)^b^	170 (22.9)	216 (30.0)^a^	
**Satisfying friendships with a circle of classmates**						
*Yes/Not yet*		2331 (94.6)	998 (98.6)^a^	674 (91.7)^b^	659 (91.8)^b^	**<0.001**
*No*		134 (5.4)	14 (1.4)^b^	61 (8.3)^a^	59 (8.2)^a^	
**Climate among classmates**						
*Friendly/Competitive but stimulating/No opinion*		2094 (83.9)	1008(97.3)^a^	584 (79.0)^b^	502 (69.6)^b^	**<0.001**
*Competitive and hostile*		402 (16.1)	28 (2.7)^b^	155 (21.0)^a^	219 (30.4)^a^	
**Grade average and satisfaction for grade average**						
*High average*:	*Satisfied*	634 (43.6)	-	283 (38.4)^b^	351 (48.9)^a^	**<0.001**
	*Not satisfied*	276 (19.0)	-	136 (18.5)	140 (19.5)	
*Low/medium average*:	*Satisfied*	153 (10.5)	-	82 (11.1)	71 (9.9)	
	*Not satisfied*	392 (26.9)	-	236 (32.0)^a^	156 (21.7)^b^	
**Grade average and being on time with exams**						
*High average*:	*On time*	753 (51.6)	-	352 (47.6)^b^	401 (53.3)^a^	**<0.001**
	*Not on time*	158 (10.8)	-	68 (9.2)^b^	90 (12.5)^a^	
*Low/medium average*:	*On time*	305 (20.9)	-	192 (25.9)^a^	113 (15.7)^b^	
	*Not on time*	244 (16.7)	-	128 (17.3)	116 (16.1)	
***Thinking that medical school hinders*:**°						
**Exercising**^#^		1132 (45.3)	388 (37.5)^b^	372 (50.1)^a^	372 (51.4)^a^	**<0.001**
**Having hobbies**^#^		1314 (52.5)	350 (33.8)^b^	466 (62.7)^a^	498 (68.8)^a^	**<0.001**
**Seeing friends**^#^		1077 (43.1)	328 (31.7)^b^	360 (48.5)^a^	389 (53.7)^a^	**<0.001**
**Sleeping properly**^#^		1009 (40.3)	315 (30.5) ^a^	355 (47.8)^a^	339 (46.8)^a^	**<0.001**
**Resting and relaxing**^#^		1206 (48.2)	403 (39.0)^b^	405 (54.5)^a^	398 (55.0)^a^	**<0.001**
***Having chosen medical school for*:**°						
**High-gain opportunities**^#^		252 (10.1)	80 (7.7)^b^	81 (10.9)	91 (12.6)^a^	**0.003**
**Employment opportunities/social status**^#^		736 (29.4)	282 (27.2)	219 (29.6)	235 (32.4)	0.060
**Personal/family experience of disease**^#^		415 (16.6)	194 (18.7)^a^	118 (15.9)	103 (14.2)^b^	**0.037**
**Acquaintance/relative MD influence**^#^		163 (6.5)	59 (5.7)	44 (5.9)	60 (8.3)	0.072
**Helping people who suffer**^#^		1290 (51.5)	603 (58.1)^a^	359 (48.4)^b^	328 (45.2)^b^	**<0.001**
**Interest in human relations**^#^		922 (36.8)	393 (37.9)	261 (35.2)	268 (37.0)	0.513
**Intellectual curiosity**^#^		1130 (45.1)	424 (40.9)^b^	359 (48.4)^a^	347 (47.9)	**0.001**
**Imposition by parents/relatives**^#^		14 (0.6)	2 (0.2)	5 (0.7)	7 (1.0)	0.089
***Worries about the future*:**°						
**No, future is stimulating**^#^		611 (24.5)	397 (38.5)^a^	121 (16.3)^b^	93 (12.8)^b^	**<0.001**
**No, not thinking about future**^#^		158 (6.3)	90 (8.7)^a^	54 (7.3)	14 (1.9)^b^	**<0.001**
**Yes, not measured up to the profession**^#^		1164 (46.6)	414 (40.2)^b^	378 (50.9)^a^	372 (51.2)^a^	**<0.001**
**Yes, about the specialty choice**^#^		522 (20.9)	80c (7.8)^b^	192 (25.9)^a^	250 (34.4)^a^	**<0.001**
**Yes, about specialty/job limited chances**^#^		1157 (46.3)	322 (31.3)^b^	372 (50.1)^a^	463 (63.8)^a^	**<0.001**

n = sample size.

Figures are expressed as number (N) and column percentages (%).

* p-value obtained via chi-squared test between each variable and years of course (except for age: Kruskal-Wallis test performed).

** Figures are expressed as median and interquartile range in brackets.

^a^ adjusted residual >1.96.

^b^ adjusted residual < -1.96.

^#^ possible options: “No” and “Yes”. “Yes” reported in table.

° possibility to select more options.

### Depressive symptoms

The median BDI-II score was 9 (IQR = 11). Among students with DS, the median was 20 (IQR = 8), while it was 6 among students without DS (IQR = 6). DS prevalence was 29.5% (95% CI: 27.8–31.3%). Specifically, 14.0% had mild depression, 11.1% had moderate depression, and 4.5% had severe depression. DS presence was not equally frequent across years: 4^th^-year and 6^th^-year students had higher frequencies (36.9% and 36.6%), and 1^st^-year students had lower frequencies (26.6%) (p<0.001). Students who took psychiatric medications accounted for 10.5% of people with DS, showing a significant association (p<0.001) confirmed by chi-squared analyses stratified by year. Students seeing a psychologist/psychiatrist accounted for 12.1% of people with DS, showing a significant association (p<0.001), and comparable associations were reported after stratifying by year. Among participants without DS, 3.7% took psychiatric medications, and 3.6% were seeing a psychologist/psychiatrist. No significant associations were identified between DS and hypothetical counselling use. Other data are presented in Table 2.

**Table 2:** Associations between depressive symptom presence and characteristics of the sample: Overall and stratified by year of course chi-squared analyses (2018).

Characteristic		Overall			1^st^ year			4^th^ year			6^th^ year		
		*Depressive symptoms*			*Depressive symptoms*			*Depressive symptoms*			*Depressive symptoms*		
		No	Yes		No	Yes		No	Yes		No	Yes	
		n = 1688	n = 708	p^1^	n = 804	n = 188	p^2^	n = 428	n = 261	p^3^	n = 456	n = 259	p^4^
		%	%		%	%		%	%		%	%	
Age*		22 (5)	23 (3)	**<0.001**	19 (1)	20 (1)	**0.007**	22 (1)	22 (1)	**0.004**	24 (1)	25 (1)	**0.009**
**Gender**													
*Male*		42.9^a^	28.2^b^	**<0.001**	41.6^a^	30.3^b^	**0.004**	46.7^a^	23.3^b^	**<0.001**	41.5^a^	31.7^b^	**0.009**
*Female*		57.1^b^	71.8^a^		58.4^b^	69.7^a^		53.3^b^	76.7^a^		58.5^b^	68.3^a^	
**Medical school geographical area**													
*Northern Italy*		40.9	39.3	0.439	40.9	38.3	0.096	43.2	39.5	0.221	38.6	39.8	0.868
*Central Italy*		21.1	19.9		23.9	18.6		17.3	22.6		19.7	18.1	
*Southern Italy*		38.0	40.8		35.2	43.1		39.5	37.9		41.7	42.1	
**Nationality**													
*Italian*		98.9	98.4	0.393	98.2	97.3	0.415	99.3	98.8	0.536	99.6	98.8	0.268
*Other*		1.1	1.6		1.8	2.7		0.7	1.2		0.4	1.2	
**Relationship status**													
*Single*		48.5	47.6	0.671	55.7	52.4	0.418	46.4	49.2	0.466	38.0	42.5	0.243
*Involved*		51.5	52.4		44.3	47.6		53.6	50.8		62.0	57.5	
**Sexual orientation**													
*Heterosexual*		88.8^a^	80.3^b^	**<0.001**	89.9^a^	75.7^b^	**<0.001**	88.5	81.7	0.090	87.2	82.1	0.034
*Homosexual*		1.6	2.1		1.0	1.6		1.9	3.1		2.4	1.6	
*Bisexual*		9.2^b^	16.2^a^		8.8^b^	21.1^a^		9.1	14.8		9.9	14.0	
*Asexual*		0.4^b^	1.4^a^		0.3^b^	1.6^a^		0.5	0.4		0.4^b^	2.3^a^	
**Living condition**													
*Alone*		5.5^a^	2.5^b^	**0.004**	4.9	2.7	0292	5.4	2.3	0.147	6.6	2.7	0.067
*With parents/relatives*		50.6	49.6		53.2	51.1		46.4	48.3		49.9	49.8	
*With partner/housemates*		44.0	47.9		41.9	46.3		48.2	49.4		43.5	47.5	
**Family cohesion**													
*Very poor/poor/excessive*		7.9^b^	17.6^a^	**<0.001**	7.1^b^	17.6^a^	**<0.001**	7.3^b^	17.3^a^	**<0.001**	9.9^b^	17.8^a^	**<0.001**
*Good*		34.2^b^	45.8^a^		3.7^b^	44.4^a^		37.9^b^	48.1^a^		36.7^b^	44.4^a^	
*Excellent*		57.9^a^	36.7^b^		62.2^a^	38.^b^		54.8^a^	34.6^b^		53.4^a^	37.8^b^	
**Working because of income need**													
*No job/job not for income need*		96.7^a^	93.2^b^	**<0.001**	97.4^a^	94.1^b^	**0.024**	96.3	93.5	0.098	96.0^a^	92.2^b^	**0.030**
*Yes*		3.3^b^	6.8^a^		2.6^b^	5.9^a^		3.7	6.5		4.0^b^	7.8^a^	
**Distance from home and economic status**													
											
*Not far from home*:	*Good status*	36.1	33.2	**<0.001**	36.8	31.6	**<0.001**	33.6	30.2	**<0.001**	37.2	37.4	**<0,001**
	*Poor status*	3.3^b^	7.0^a^		3.2^b^	7.0^a^		3.1^b^	6.6^a^		3.8^b^	7.4^a^	
*Far from home*:	*Good status*	57.1^a^	48.7^b^		55.5	48.7		60.1^a^	50.0^b^		57.1^a^	47.5^b^	
	*Poor status*	3.5^b^	11.1^a^		4.6^b^	12.8^a^		3.3^b^	13.2^a^		2.0^b^	7.8^a^	
**Having at least one hobby**^#^		74.9^a^	58.0^b^	**<0.001**	77.8^a^	63.6^b^	**<0.001**	72.6^a^	53.6^b^	**<0.001**	71.9^a^	58.3^b^	**<0.001**
**Exercising less than 90 min weekly**^#^		54.8^b^	63.3^a^	**<0.001**	58.7^b^	70.2^a^	**0.012**	48.4^b^	58.2^a^	**0.014**	53.8^b^	63.3^a^	**0.004**
**1**^**st**^**/2**^**nd**^ **degree relatives with psychiatric disorders**^#^		21.0^b^	32.3^a^	**<0.001**	17.9^b^	32.3^a^	**<0.001**	21.4^b^	31.8^a^	**0.002**	26.0	32.8	0.054
											
**Suicides/suicide attempts among 1**^**st**^**/2**^**nd**^ **degree relatives**^#^		4.9^b^	7.6^a^	**0.009**	4.9	8.0	0.085	4.0^b^	8.1^a^	**0.022**	5.9	6.9	0.591
											
**Having a chronic disease**^#^		5.9^b^	11.0^a^	**<0.001**	2.9^b^	6.9^a^	**0.008**	5.8	8.1	0.249	11.2^b^	17.0^a^	**0.029**
**Taking stimulants to improve academic performance**^#^		4.1^b^	6.6^a^	**0.008**	3.6	4.3	0.672	5.1^b^	9.2^a^	**0.039**	3.9	5.8	0.259
											
**Judging the choice of medical school**													
*Positively*		87.6^a^	61.0^b^	**<0.001**	90.6^a^	74.5^b^	**<0.001**	88.7^a^	61.7^b^	**<0.001**	81.2^a^	50.6^b^	**<0.001**
*Negatively/No opinion*		12.4^b^	39.0^a^		9.4^b^	25.5^a^		11.3^b^	38.3^a^		18.8^b^	49.4^a^	
**Satisfying friendships with a circle of classmates**													
											
*Yes/Not yet*		96.6^a^	90.5^b^	**<0.001**	98.9	97.2	0.095	96.0^a^	87.4^b^	**<0.001**	93.4^a^	89.0^b^	**0.043**
*No*		3.4^b^	9.5^a^		1.1	2.8		4.0^b^	12.6^a^		6.6^b^	11.0^a^	
**Climate among classmates**													
*Friendly/Competitive but stimulating/No opinion*		89.2^a^	71.3^b^^b^	**<0.001**	98.5^a^	93.1^b^	**<0.001**	86.2^a^	68.0^b^	**<0.001**	75.7^a^	58.8^b^	**<0.001**
*Competitive and hostile*		10.8^b^	28.7^a^		1.5^b^	6.9^a^		13.8^b^	32.0^a^		24.3^b^	41.2^a^	
**Grade average and satisfaction for grade average**													
											
*High average*:	*Satisfied*	51.8^a^	30.8^b^	**<0.001**	-			47.2^a^	24.8^b^	**<0.001**	56.1^a^	36.7^b^	**<0.001**
	*Not satisfied*	14.4^b^	25.3^a^		-			13.4^b^	24.0^a^		15.4^b^	26.6^a^	
*Low/medium average*:	*Satisfied*	12.3^a^	7.7^b^		-			13.2^a^	7.8^b^		11.4	7.7	
	*Not satisfied*	21.5^b^	36.2^a^		-			26.2^b^	43.4^a^		17.1^b^	29.0^a^	
**Grade average and being on time with exams**													
											
*High average*:	*On time*	60.1^a^	37.9^b^	**<0.001**	-			56.6^a^	33.2^b^	**<0.001**	63.5^a^	42.6^b^	**<0.001**
	*Not on time*	5.9^b^	18.0^a^		-			4.0^b^	15.4^a^		7.7^b^	20.5^a^	
*Low/medium average*:	*On time*	23.7^a^	16.8^b^		-			30.5^a^	21.2^b^		17.3	12.4	
	*Not on time*	10.3^b^	27.3^a^		-			8.9^b^	30.1^a^		11.5^b^	24.4^a^	
***Thinking that medical school hinders*:°**													
**Exercising**^#^		42.6^b^	52.5^a^	**<0.001**	36.4	43.6	0.065	46.7^b^	56.9^a^	**0.010**	49.7	54.5	0.218
**Having hobbies**^#^		46.3^b^	68.5^a^	**<0.001**	29.5^b^	53.7^a^	**<0.001**	55.4^b^	75.4^a^	**<0.001**	67.3	72.4	0.156
**Seeing friends**^#^		39.0^b^	53.6^a^	**<0.001**	29.8^b^	41.0^a^	**0.003**	44.2^b^	57.3^a^	**0.001**	50.5^b^	59.1^a^	**0.027**
**Sleeping properly**^#^		33.2^b^	57.4^a^	**<0.001**	26.9^b^	44.7^a^	**<0.001**	37.9^b^	64.2^a^	**<0.001**	40.0^b^	59.9^a^	**<0.001**
**Resting and relaxing**^#^		43.1^b^	61.8^a^	**<0.001**	35.9^b^	52.7^a^	**<0.001**	47.7^b^	68.1^a^	**<0.001**	51.4^b^	62.3^a^	**<0.001**
***Having chosen medical school for*:°**													
**High-gain opportunities**^#^		9.1^b^	12.9^a^	**0.005**	7.4	10.7	0.129	10.6	11.1	0.830	10.8^b^	16.3^a^	**0.034**
**Employment opportunities/social status**^#^		29.5	29.0	0.825	26.8	25.1	0.640	30.1	30.7	0.883	33.6	30.2	0.352
											
**Personal/family experience of disease**^#^		15.9	19.0	0.064	18.0^a^	24.1^b^	0.056	13.6^b^	19.5^a^	**0.040**	14.3	14.7	0.872
**Acquaintance/relative MD influence**^#^		6.5	6.4	0.923	4.9	8.6	**0.047**	6.8	4.6	0.232	9.0	6.6	0.256
**Helping people who suffer**^#^		53.3^a^	47.2^b^	**0.006**	58.6	56.7	0.632	50.1	45.2	0.212	46.8	42.2	0.239
**Interest in human relations**^#^		36.4	37.5	0.595	38.8	33.7	0.196	33.2	37.9	0.205	35.2	39.9	0.206
**Intellectual curiosity**^#^		54.6	55.4	0.738	41.1	41.7	0.888	49.6	45.6	0.302	48.8	45.7	0.433
**Imposition** ^b^**y parents/relatives**^#^		0.4	1.0	0.93	0.1	0.5	0.261	0.2	1.5	0.052	1.1	0.8	0.674
***Worries about the future*:°**													
**No, future is stimulating**^#^		31.4^a^	8.1^b^	**<0.001**	43.9^a^	15.0^b^	**<0.001**	22.8^a^	6.5^b^	**<0.001**	17.5^a^	4.6^b^	**<0.001**
**No, not thinking about future**^#^		7.8^a^	2.8^b^	**<0.001**	10.0^a^	3.2^b^	**0.003**	9.4^a^	4.2^b^	**0.012**	2.4	1.2	0.245
**Yes, not measured up to the profession**^#^		37.9^b^	67.8^a^	**<0.001**	33.9^b^	66.8^a^	**<0.001**	40.6^b^	69.0^a^	**<0.001**	42.3^b^	67.2^a^	**<0.001**
											
**Yes, about the specialty choice**^#^		16.8^b^	30.0^a^	**<0.001**	6.1^b^	15.0^a^	**<0.001**	21.4^b^	31.0^a^	**0.005**	31.4^b^	39.8^a^	**0.023**
**Yes, about specialty/job limited chances**^#^		40.6^b^	60.4^a^	**<0.001**	27.3^b^	49.7^a^	**<0.001**	44.1^b^	59.8^a^	**<0.001**	60.5^b^	68.7^a^	**0.029**
											

n = sample size.

Figures are expressed as column percentages (%).

* Figures are expressed as median and interquartile range in brackets.

^1^ p-value obtained via chi-squared test between each variable and depressive symptom presence in the whole sample (except for age: Mann-Whitney U test performed).

^2^ p-value obtained via chi-squared test between each variable and depressive symptoms in the 1^st^ year (except for age: Mann-Whitney U test performed).

^3^ p-value obtained via chi-squared test between each variable and depressive symptoms in the 4^th^ year (except for age: Mann-Whitney U test performed).

^4^ p-value obtained via chi-squared test between each variable and depressive symptoms in the 6^th^ year (except for age: Mann-Whitney U test performed).

^a^ adjusted residual >1.96.

^b^ adjusted residual < -1.96.

^#^ possible options: “No” and “Yes”. “Yes” reported in table.

° possibility to select more options.

The whole sample regression (Table 3) showed some risk factors for DS presence, e.g., increasing age, being female, bisexual/asexual orientation, living with partner/housemates, poor economic status (worsened by living far from home), less than 90 min of weekly exercise, relatives with psychiatric disorders, personal chronic disease, judging medical school choice negatively, having unsatisfying friendships with classmates, competitive and hostile climate among classmates, thinking that medical school hinders activities and being worried about not measuring up to the profession. Good/excellent family cohesion, hobbies, intellectual curiosity as a career motivation and no worries about the future were protective factors. Differences in predictors among years of course were investigated in stratified models (Table 3).

**Table 3:** Predictors for depressive symptom presence: Overall and stratified by year of course multivariable logistic regression models (2018).

Predictors for depressive symptoms presence		Overall*		1st year		4th year		6th year	
		adjOR (95% CI)	p	adjOR (95% CI)	p	adjOR (95% CI)	p	adjOR (95% CI)	p
**Age**		1.09 (1.03–1.15)	**0.004**	1.09 (0.96–1.23)	0.173	1.11 (0.95–1.30)	0.196	1.05 (0.97–1.15)	0.227
**Gender**									
*Male*		Ref.		Ref.		Ref.		Ref.	
*Female*		1.49 (1.17–1.90)	**0.001**	1.27 (0.82–1.95)	0.282	2.80 (1.72–4.56)	**<0.001**	1.04 (0.69–1.56)	0.860
**Medical school geographical area**									
*Northern Italy*		Ref.		Ref.		Ref.			
*Central Italy*		0.89 (0.65–1.20)	0.441	0.84 (0.48–1.48)	0.547	2.33 (1.27–4.27)	**0.006**		
*Southern Italy*		1.35 (1.06–1.74)	**0.017**	2.17 (1.39–3.41)	**0.001**	1.41 (0.87–2.27)	0.160		
**Nationality**									
*Italian*				Ref.					
*Other*				3.80 (0.88–16.42)	0.074				
**Relationship status**									
*Single*		Ref.				Ref.			
*Involved*		0.82 (0.65–1.03)	0.085			0.70 (0.46–1.07)	0.100		
**Sexual orientation**									
*Heterosexual*		Ref.		Ref.		Ref.			
*Homosexual*		1.53 (0.68–3.47)	0.304	1.59 (0.27–9.53)	0.611	9.39 (2.41–36.55)	**0.001**		
*Bisexual*		1.70 (1.22–2.36)	**0.002**	2.96 (1.70–5.15)	**<0.001**	1.41 (0.74–2.70)	0.298		
*Asexual*		3.84 (1.12–13.09)	**0.032**	16.47 (1.63–165.96)	**0.017**	0.097 (0.00–6.56)	0.278		
**Living condition**									
*Alone*		Ref.						Ref.	
*With parents/relatives*		1.76 (0.91–3.42)	0.095					1.92 (0.64–5.81)	0.246
*With partner/housemates*		1.98 (1.05–3.75)	**0.036**					3.21 (1.10–9.42)	**0.034**
**Family cohesion**									
*Very poor/poor/excessive*		Ref.		Ref.		Ref.		Ref.	
*Good*		0.65 (0.46–0.93)	**0.019**	0.59 (0.30–1.13)	0.109	0.55 (0.28–1.11)	0.095	0.66 (0.37–1.20)	0.172
*Excellent*		0.41 (0.29–0.59)	**<0.001**	0.31 (0.16–0.60)	**<0.001**	0.33 (0.16–0.66)	**0.002**	0.47 (0.26–0.85)	**0.013**
**Distance from home and economic status**									
							
*Not far from home*:	*Good status*	Ref.				Ref.		Ref.	
	*Poor status*	1.94 (1.17–3.22)	**0.010**			1.88 (0.68–5.25)	0.226	2.66 (1.07–6.58)	**0.035**
*Far from home*	*Good status*	0.84 (0.60–1.18)	0.320			0.79 (0.50–1.24)	0.294	0.72 (0.40–1.30)	0.278
	*Poor status*	2.64 (1.58–4.41)	**<0.001**			3.28 (1.31–8.26)	**0.012**	3.05 (0.99–9.31)	0.051
**Having at least one hobby**^#^		0.65 (0.51–0.82)	**<0.001**	0.58 (0.38–0.89)	**0.012**	0.53 (0.34–0.82)	**0.004**	0.62 (0.42–0.93)	**0.019**
**Exercising less than 90 min weekly**^#^		1.35 (1.07–1.70)	**0.010**						
**1**^**st**^**/2**^**nd**^ **degree relatives with psychiatric disorders**^#^		1.41 (1.10–1.81)	**0.007**	1.48 (0.94–2.33)	0.094			1.59 (1.05–2.42)	**0.028**
							
**Having a chronic disease**^#^		1.48 (1.00–2.19)	**0.048**						
**Judging the choice of medical school**									
*Positively*		Ref.		Ref.		Ref.		Ref.	
*Negatively/No opinion*		2.76 (2.12–3.59)	**<0.001**	2.42 (1.42–4.11)	**0.001**	2.83 (1.70–4.72)	**<0.001**	3.18 (2.10–4.82)	**<0.001**
**Satisfying friendships with a circle of classmates**									
							
*Yes/Not yet*		Ref.				Ref.			
*No*		1.71 (1.06–2.75)	**0.027**			2.02 (0.89–4.55)	0.092		
**Climate among classmates**									
*Friendly/Competitive but stimulating/No opinion*		Ref.		Ref.				Ref.	
							
*Competitive and hostile*		2.10 (1.58–2.81)	**<0.001**	5.28 (2.00–13.95)	**0.001**			2.03 (1.35–3.04)	**0.001**
**Grade average and satisfaction for grade average**									
							
*High average*:	*Satisfied*	-		-		Ref.		Ref.	
	*Not satisfied*	-		-		2.01 (1.09–3.73)	**0.026**	1.80 (1.09–2.98)	**0.022**
*Low/medium average*:	*Satisfied*	-		-		3.16 (1.31–7.65)	**0.011**	2.07 (0.92–4.67)	0.079
	*Not satisfied*	-		-		5.68 (2.96–10.89)	**<0.001**	3.24 (1.85–5.67)	**<0.001**
**Grade average and being on time with exams**									
							
*High average*:	*On time*	-		-		Ref.		Ref.	
	*Not on time*	-		-		4.40 (1.92–10.09)	**<0.001**	2.15 (1.19–3.89)	**0.012**
*Low/medium average*:	*On time*	-		-		2.64 (1.46–4.80)	**0.001**	2.02 (1.09–3.75)	**0.025**
	*Not on time*	-		-		5.68 (2.96–10.89)	**<0.001**	3.24 (1.85–5.67)	**<0.001**
***Thinking that medical school hinders*:°**									
**Having hobbies**^#^		1.31 (1.03–1.67)	**0.027**	1.78 (1.18–2.69)	**0.006**	1.65 (1.05–2.59)	**0.032**		
**Sleeping properly**^#^		1.79 (1.41–2.26)	**<0.001**			2.26 (1.47–3.47)	**<0.001**	1.67 (1.14–2.45)	**0.008**
**Resting and relaxing**^#^		1.29 (1.02–1.64)	**0.034**	1.61 (1.08–2.41)	**0.021**				
***Having chosen medical school for*:°**									
**Employment opportunities/social status**^#^								1.01 (0.66–1.54)	0.973
**Acquaintance/relative MD influence**^#^				2.07 (0.97–4.44)	0.063				
**Helping people who suffer**^#^		0.82 (0.65–1.03)	0.091			0.79 (0.52–1.20)	0.265		
**Intellectual curiosity**^#^		0.74 (0.59–0.93)	**0.011**						
***Worries about the future*:°**									
**No, future is stimulating**^#^		0.48 (0.33–0.70)	**<0.001**					0.52 (0.25–1.07)	0.077
**No, not thinking about future**^#^		0.50 (0.27–0.89)	**0.019**						
**Yes, not measured up to the profession**^#^		1.97 (1.52–2.55)	**<0.001**	3.15 (2.06–4.81)	**<0.001**	2.27 (1.46–3.54)	**<0.001**	2.00 (1.33–3.01)	**0.001**
**Yes, about the specialty choice**^#^				1.60 (0.99–2.59)	0.058				
**Yes, about specialty/job limited chances**^#^				2.16 (1.436–3.26)	**<0.001**				

Figures are expressed as adjusted odds ratios (adjOR) and 95% confidence intervals (CIs).

All models included age and gender. Final multivariable logistic regression models are presented in this table.

*Variables entered the first step (backward stepwise method)*: school geographical area, nationality, relationship status, sexual orientation, living condition, family cohesion, working condition, distance from home and economic status, having a hobby, practising sport, family history of psychiatric disorders and suicides/attempts, personal chronic disease, stimulant consumption, medical school choice judgement, friendship satisfaction, classmates climate, all hindrances by medical school, all career motivations and all worries about future. Grade average satisfaction and being on time with exams were entered only in the 4^th^ and 6^th^ year analyses.

* this final model included year of course (non-significant).

^#^ possible options: “No” and “Yes”. “No” considered the reference level.

° possibility to select more options.

Moreover, some risk factors were more frequent in participants far from home, e.g., 62.5% exercised less than 90 min weekly, and 54.5% thought that medical school hindered hobbies, while in students not far from home, these proportions were 49.0% and 49.9% (p<0.001, p = 0.025), respectively. In the 1^st^ year, being far from home was associated with negatively judging the medical school choice, with a 16.1% prevalence in students far from home and 7.8% in students not far from home (p<0.001).

### BDI-II subscales

Affective, cognitive and somatic subscale scores can potentially range from 0–15, 0–21, and 0–27, respectively. Overall, the affective subscale had a median score of 1 (IQR = 3), the cognitive subscale had a median score of 2 (IQR = 4) and the somatic subscale had a median score of 5 (IQR = 5). Among students with DS, the affective subscale had a median of 4 (IQR = 2), the cognitive subscale of 7 (IQR = 5) and the somatic subscale of 10 (IQR = 4). Kruskal-Wallis tests reported different distributions across years of study for each subscale (p<0.001 in all cases). Indeed, the medians for the affective subscale in the 1^st^, 4^th^ and 6^th^ years, respectively, were 1, 2 and 2 (IQR = 2, 3, 3); the medians for the cognitive subscale were 1, 3 and 3 (IQR = 4, 5, 5); and the medians for the somatic subscale were 4, 6 and 6 (IQR = 5, 5, 6). Mann-Whitney tests showed differences across genders for cognitive and somatic subscales (p<0.001 in both cases) but not for the affective subscale (p = 0.061). The median for the cognitive subscale was 2 for males and 3 for females (IQR = 4, 5), and the median for the somatic subscale was 4 for males and 6 for females (IQR = 5, 6). The regressions performed to assess differences in predictors for each subscale are reported in Table 4.

**Table 4:** Beck Depression Inventory-II (BDI-II) subscales: Multivariable linear regression models (2018).

Predictors		Affective subscale		Cognitive subscale		Somatic subscale	
		adjB (95% CI)	p	adjB (95% CI)	p	adjB (95% CI)	p
**Age**		0.03 (-0.00; 0.06)	0.070	0.05 (-0.01; 0.10)	0.100	0.05 (-0.01; 0.12)	0.113
**Gender**							
*Male*		Ref.		Ref.		Ref.	
*Female*		-0.12 (-0.24; 0.01)	0.073	0.33 (0.12; 0.54)	**0.002**	0.65 (0.40; 0.90)	**<0.001**
**Year of course**							
*1*^*st*^		Ref.		Ref.		Ref.	
*4*^*th*^		-0.01 (-0.19; .17)	0.912	0.22 (0.08; 0.51)	0.144	0.33 (-0.03; 0.68)	0.071
*6*^*th*^		-0.16 (-0.39; 0.08)	0.186	-0.26 (-0.65; 0.13)	0.184	-0.48 (-0.95; 0.01)	**0.046**
**Medical school geographical area**							
*Northern Italy*		Ref.		Ref.		Ref.	
*Central Italy*		-0.04 (-0.19; 0.12)	0.639	0.01 (-0.26; 0.27)	0.993	0.10 (-0.22; 0.42)	0.554
*Southern Italy*		0.28 (0.15; 0.41)	**<0.001**	0.34 (0.12; 0.56)	**0.002**	0.56 (0.29; 0.82)	**<0.001**
**Relationship status**							
*Single*		Ref.		Ref.			
*Involved*		-0.30 (-0.42; -0.19)	**<0.001**	-0.36 (-0.55; -0.16)	**<0.001**		
**Sexual orientation**							
*Heterosexual*		Ref.		Ref.		Ref.	
*Homosexual*		0.18 (-0.27; 0.62)	0.437	1.07 (0.33; 1.80)	**0.004**	-0.08 (-0.97; 0.81)	0.857
*Bisexual*		0.32 (0.13; 0.51)	**0.001**	0.56 (0.24 0.89)	**0.001**	0.78 (0.38; 1.17)	**<0.001**
**Living condition**							
*Alone*		Ref.		Ref.			
*With parents/relatives*		-0.01 (-0.31; 0.29)	0.947	0.15 (-0.36; 0.66)	0.566		
*With partner/housemates*		-0.15 (-0.43; 0.14)	0.311	-0.11 (-0.59; 0.37)	0.646		
**Family cohesion**							
*Very poor/poor/excessive*		Ref.		Ref.		Ref.	
*Good*		-0.42 (-0.63; -0.21)	**<0.001**	-0.56 (-0.92; -0.20)	**0.002**	-0.36 (-0.78; 0.07)	0.098
*Excellent*		-0.68 (-0.89; -0.47)	**<0.001**	-1.14 (-1.49; -0.79)	**<0.001**	-0.72 (-1.14; -0.30)	**0.001**
**Working because of income need**							
*No job/job not for income need*		Ref.				Ref.	
*Yes*		0.37 (0.05; 0.70)	**0.024**			0.65 (-0.03; 1.31)	0.060
Distance from home and economic status*							
*Not far from home*:	*Good status*	Ref.				Ref.	
	*Poor status*	0.49 (0.16; 0.82)	**0.003**	0.21 (-0.31; 0.73)	0.43	1.48 (0.82; 2.13)	**<0.001**
*Far from home*	*Good status*	0.06 (-0.11; 0.23)	0.478	0.14 (-0.15; 0.43)	0.331	-0.15 (-0.40; 0.10)	0.237
	*Poor status*	0.47 (0.17; 0.77)	**0.002**	0.91 (0.38; 1.43)	**0.001**	1.07 (0.79; 1.64)	**<0.001**
**Having at least one hobby**^#^		-0.36 (-0.49; -0.23)	**<0.001**	-0.24 (-0.46; -0.02)	**0.032**	-0.68 (-0.94; -0.42)	**<0.001**
**Exercising less than 90 min weekly**^#^		0.10 (-0.01; 0.22)	0.086	0.21 (0.01; 0.41)	**0.043**	0.44 (0.20; 0.69)	**<0.001**
**1**^**st**^**/2**^**nd**^ **degree relatives with psychiatric disorders**^#^		0.21 (0.07; 0.34)	**0.003**	0.38 (0.15; 0.62)	**0.001**	0.25 (-0.03; 0.53)	0.079
**Having a chronic disease**^#^		0.21 (-0.01; 0.43)	0.066	0.40 (0.02; 0.79)	**0.039**	0.78 (0.31; 1.24)	**0.001**
**Judging the choice of medical school**							
*Positively*		Ref.		Ref.		Ref.	
*Negatively/No opinion*		0.93 (0.77; 1.08)	**<0.001**	1.46 (1.20; 1.73)	**<0.001**	1.19 (0.87; 1.50)	**<0.001**
**Satisfying friendships with a circle of classmates**							
*Yes/Not yet*		Ref.		Ref.		Ref.	
*No*		0.76 (0.46; 1.06)	**<0.001**	1.16 (0.61; 1.70)	**<0.001**	0.62 (0.02; 1.23)	**0.045**
**Climate among classmates**							
*Friendly/Competitive but stimulating/No opinion*		Ref.		Ref.		Ref.	
*Competitive and hostile*		0.65 (0.48; 0.82)	**<0.001**	1.11 (0.82; 1.40)	**<0.001**	1.26 (0.91; 1.61)	**<0.001**
***Thinking that medical school hinders*:°**							
**Exercising**^#^						0.23 (-0.03; 0.48)	0.069
**Having hobbies**^#^		0.27 (0.15; 0.40)	**<0.001**	0.33 (0.12; 0.54)	**0.002**	0.38 (0.12; 0.64)	**0.005**
**Sleeping properly**^#^		0.35 (0.23; 0.48)	**<0.001**	0.55 (0.34; 0.76)	**<0.001**	1.34 (1.08; 1.60)	**<0.001**
**Resting and relaxing**^#^		0.27 (0.05; 0.44)	**<0.001**			0.82 (0.57; 1.08)	**<0.001**
***Having chosen medical school for*:°**							
**High-gain opportunities**^#^		0.24 (0.05; 0.44)	**0.013**				
**Employment opportunities/social status**				-0.18 (-0.39; 0.04)	0.112		
**Helping people who suffer**^#^		-0.27 (-0.39; -0.15)	**<0.001**				
**Intellectual curiosity**^#^		-0.13 (-0.25; -0.01)	**0.033**			-0.32 (-0.55; -0.08)	**0.009**
**Worries about the future:°**							
**No, future is stimulating**^#^						-0.46 (-0.85; -0.07)	**0.021**
**No, not thinking about future**^#^						-0.76 (-1.34; -0.22)	**0.006**
**Yes, not measured up to the profession**^#^		0.49 (0.37; 0.61)	**<0.001**	1.39 (1.18; 1.89)	**<0.001**	0.76 (0.46; 1.07)	**<0.001**
**Yes, about the specialty choice**^#^		0.24 (0.09; 0.39)	**0.002**	0.33 (0.086; 0.59)	**0.010**		
**Yes, about specialty/job limited chances**^#^		0.27 (0.14; 0.39)	**<0.001**	0.38 (0.17; 0.58)	**0.001**	0.45 (0.15; 0.75)	**0.003**

**Affective subscale items:** sadness, pessimism, loss of pleasure, suicidal thoughts or wishes, loss of interest.

**Cognitive subscale items:** past failure, guilty feelings, punishment feelings, self-dislike, self-criticalness, indecisiveness, worthlessness.

**Somatic subscale items:** crying, agitation, loss of energy, changes in sleeping pattern, irritability, changes in appetite, concentration difficulty, tiredness or fatigue, loss of interest in sex.

Figures are expressed as adjusted unstandardized coefficient B (adjB) and 95% confidence interval (CI).

All models included age and gender. Final multivariable linear regression models are presented in this table.

*Variables entered the first step (backward elimination method)*: year, school geographical area, nationality, relationship status, sexual orientation, living condition, family cohesion, working condition, distance from home and economic status, having a hobby, exercising, family history of psychiatric disorders and suicides/attempts, personal chronic disease, stimulant consumption, medical school choice judgement, friendship satisfaction, classmates climate, all hindrances by medical school, all career motivations and all worries about future.

^#^ possible options: “No” and “Yes”. “No” considered the reference level.

## Discussion

The PRIMES aimed to evaluate DS prevalence among Italian medical students and assess risk and protective factors to have a basis for planning preventive interventions.

Our DS prevalence was 29.5%, consistent with the prevalence among medical students worldwide (27.0%, 95% CI: 24.7–29.5%) but higher than that among European students (20.1%, 95% CI: 15.8–25.1%) [3]. The PRIMES confirmed that medical students are a high-risk population: PASSI, one of the major Italian surveys, reported a DS prevalence of 4.6% (95% CI, 4.3–4.9) among people 18–34 years old [29]. Moreover, the prevalence of depressive disorders is 3.59% [95% CI, 3.34–3.89%] worldwide, 4.17% (95% CI, 3.89–4.50) in Europe and 4.11% (95% CI, 3.80–4.46%) in Italy [1]. When considering persons 20–24 years old, their prevalence is approximately 4% [1]. Although DS prevalence among medical students is evidently higher than among the general population, there is no sufficient evidence to infer whether DS are more common among medical students than among other university students [30].

Generally, the predictors were consistent with the literature. For instance, sexual minorities are high-risk populations for mental issues [14]; however, few studies have focused on this aspect of medical students’ lives, confirming that medical students belonging to sexual minorities have a greater DS risk [31]. Furthermore, a family history of psychiatric issues [32] and negative life events, e.g., personal disease [15], have been associated with depression. Moreover, depression and bipolar disorders have been widely recognized as familial conditions [16]. Financial concerns and constraints have been identified as stressors for medical students [17], and interestingly, we found higher ORs in participants far from home. Perhaps the distance could amplify one’s poor economic status, and supportive interventions for this category of students need to be planned. In addition, the transition of moving away from home has been reported as stressful [6], but it did not emerge from the PRIMES. However, the higher prevalence of certain risk factors in students far from home, e.g., low exercise, medical school being an obstacle to hobbies and negative judgement about medical school choice, might be a warning to dedicate special care to such students.

Conversely, social support seems to influence resiliency among medical students. Strong support from family, medical school staff and peers and positive perceptions of the learning climate were found to be associated with resilience and protection against mental issues [18,26], as confirmed by our findings on family cohesion, friendships with classmates and climate among classmates. Moreover, consistent with the PRIMES, physical inactivity has been reported as a risk factor for mental issues [33]. Exercise is known to be an effective intervention for depression [34], and several projects proposed exercise-related activities in medical students’ curriculum due to mental benefits [21,35]. Additionally, sports and hobbies, as extracurricular activities, can reduce stress [22]. The lack of time has been reported as a barrier to exercise among medical students [36]. Since other factors emerged from the PRIMES, e.g., thinking that medical schools hinder hobbies/sleeping/resting, investigating whether such barriers are also related to these activities would be useful. Finally, fears of not measuring up to the profession have been identified as stressors [6], and it is not surprising that not having worries about future careers was protective against DS. Even if intellectual curiosity has already been reported among the common motivations for starting medical school [24], this study is the first to report that this motivation is related to a significantly lower risk of DS. Interestingly, the PRIMES highlighted that students who negatively judged the choice of attending medical school had higher risk of DS independent of the motivations behind such choices or the worries about the future. These relationships among DS and career motivations or judgements should be further investigated.

Contrary to the relevant literature about medical students and depressive symptoms, in our study, age, being female and living with partners/housemates were associated with DS. Specifically, a recent meta-analysis found that the associations between DS and gender or age were not significant in medical students [5]. However, epidemiological data confirmed that women’s depressive risk is higher in the general population [37]. This relationship among medical students should be further investigated, and additional efforts must be concentrated on understanding the associations with age. Moreover, living with partners/housemates was expected not to be a risk factor due to the protective nature of social support [18]; therefore, it is necessary to study cohabitation aspects in more detail.

Finally, the association of DS with studying in medical schools in Southern Italy is not clear. Further analyses should be addressed to understand whether emerged differences are linked to medical schools or regional characteristics, as regions have always been different in size, geographical character, economic development and culture [38].

The regression analyses showed no outstanding differences between the overall and stratified models. DS presence was not equally frequent across years, but the year of study did not have an association with DS in the regression model. This difference might be explained by other model variables rather than year per se. Indeed, a recent meta-analysis concluded that the association of DS with year of study is unclear [10]. Nevertheless, the frequencies of risk and protective factors are differently distributed among years (Table 1); therefore, different preventive strategies may be advisable. Furthermore, the 4^th^- and 6^th^-year models included grade average, satisfaction for grade average and being on time with exams, which affected DS presence. In this regard, it has been reported that pass/fail evaluation systems can improve well-being without negatively affecting academic results, and tiered systems may hinder the ability to achieve a desired specialty [39].

Our subscale analyses underlined some differences in predictors among BDI-II dimensions; however, few studies focused on subscales, and few comparable studies on medical students exist. The affective cluster was found to be associated with leisure and colleagues, suggesting that the development of certain depression types is influenced by social factors, while the somatic cluster was related to studies and patients [40]. In the PRIMES, the affective subscale differed mainly for financial items, and the somatic differed mainly for protective effects of not being worried for future, thus highlighting other possible mechanisms below. These analyses were performed to produce hypotheses on DS clustering since most studies concentrated on total scores, but further insights are required to understand DS characteristics among medical students to develop specific plans.

Data on students taking psychiatric medications and students seeing a psychologist/psychiatrist (10.5% and 12.1% of participants with DS, respectively) showed a possible under-treatment, as reported in meta-analyses [5,10]. Rotenstein et al. found that 15.7% of depressed medical students sought treatment, and Puthran et al. reported a prevalence of 12.9% [5,10]. This phenomenon is mainly explained by fear of negative impact on career, disease denial, poor insight, lack of time, stigma related to mental services utilization and undesired interventions [10]. Moreover, only 38.9% of our sample knew about university counselling, and alarmingly, less than half reported that they would use it if necessary. Currently, mental health services for medical students are expanding, and effective interventions are under investigation [7,8]. Beyond the indispensable mental services and counselling that the university must provide [7], individually focused mindfulness-based interventions seem to be the most effective in lowering depression levels in medical students [8]. Furthermore, student well-being might be improved by mandatory exercise [35] and a pass/fail grading system [39].

This study had several strengths and limitations that must be acknowledged. Above all, the PRIMES was the first Italian multicentre study investigating DS prevalence among medical students. The multicentre design and the large sample permitted us to estimate potential predictors that had never been evaluated in this context. Nevertheless, the limitations included the cross-sectional structure, which restricts causal interpretations and makes it difficult to understand if some factors lead to depression or appear as risk factors because of the typical negative thinking of depression [41], and the self-reported measures of DS rather than structured interviews and clinical diagnoses. However, the BDI-II is one of the most commonly used instruments, with firm cutoffs that are strongly predictive of clinical alterations [3,13]. Another important limitation concerned the opportunistic sampling: no data about students who did not attend lessons or refused to participate were collected, causing a potential loss of information. Finally, although the PRIMES considered several variables to explore predictors of DS, it must be stated that other potential confounders might exist, and further analyses must be addressed to study these relationships.

In conclusion, the PRIMES showed that Italian medical students are at high risk of depression; the prevalence in our study was similar to that of medical students worldwide and higher than that of European colleagues. Medical schools must make efforts to plan and implement preventive and treatment interventions by offering counselling and working on modifiable factors to improve well-being, such as lifestyle and learning climate. Further studies should more deeply examine the causes involved and find the most effective preventive strategies.

## Supporting information

S1 Appendix PRIMES sociodemographic questionnaire (translated version). (DOCX)
