# Supplementary material for: Prevalence of depressive symptoms among Italian medical students: The multicentre cross-sectional “PRIMES” study
Source: PLoS One. 2020 Apr 17;15(4):e0231845. doi: 10.1371/journal.pone.0231845 (PMC7164645; doi:10.1371/journal.pone.0231845)
Supplement: S1 Appendix — (DOCX) [file pone.0231845.s001.docx]

**S1 Appendix. PRIMES sociodemographic questionnaire (translated version)**

| 1. **Gender**: | - Male - Female - Not binary/third gender | 1. **Age:** | \|__\|__\| years |
| --- | --- | --- | --- |
| 1. **Nationality:** | - Italian - Not Italian: ____________ | 1. **Are you studying far from home?** | - No - Yes, but I am from the same region of my University - Yes, I am from a different region than my University one.   Specify which region: _____________   - Yes, I am from another country   Specify which country: __________________________ |

1. **At present, who do you live with?**

- Alone
- With parents
- With relatives
- With partner
- With housemates
- In a dormitory

1. **How do you think the cohesion of your family is?**

- Very poor
- Poor
- Good
- Excellent
- Excessive

1. **Relationship status:**

- Single
- Involved

1. **At present, who do you feel sexually attracted to?**

- Only men
- Mainly men
- Both men and women in the same way
- Mainly women
- Only women
- Neither men nor women

1. **Do you have first/second degree relatives with diagnosed psychiatric illnesses?**

- No
- Yes. How many? |__|__|

With what disease? (*you can select* *more than one answer*)

- Schizophrenia spectrum and other psychotic disorders
- Bipolar disorders
- Depressive disorders
- Anxiety disorders
- Obsessive-compulsive disorders
- Feeding and eating disorders
- Substance-related and addictive disorders
- Personality disorders
- Other: ___________________

1. **Were there suicides and/or suicide attempts in your family (including only first/second degree relatives)?**

- No
- Yes. How many? |__|__|

1. **Do you suffer from a chronic disease?**

- No
- Yes: _____________________

1. **How would you rate your family's financial situation with respect to your needs?**

- Insufficient
- Poor
- Adequate
- Excellent

1. **Do you have a job?**

- No
- Yes, by necessity and I can almost/fully provide for myself
- Yes, by necessity but I can’t provide for myself
- Yes, but not by necessity

1. **Do you exercise?**

- Yes, more than 90 min per week
- Yes, less than 90 min per week
- Yes, occasionally, less than once a week
- No

1. **Do you have a personal passion/hobby in your spare time?**

- Yes
- No

1. **Do you think that medical school prevents you from..?**(*you can select* *more than one answer*)

- I don’t think that Medical School prevents me from doing anything
- Exercising as I wish
- Having hobbies as I wish
- Seeing friends as I wish
- Sleeping properly (quantitatively and/or qualitatively)
- Resting and relaxing as I wish

1. **Did you choose medical school mainly for..?** (*you can select* *more than one answer*)

- High gain opportunities
- Employment opportunities and social status
- Personal/family experience of disease
- Influence by an acquaintance/relative who is a medical doctor
- Helping people who suffer
- Interest in human relations
- Intellectual curiosity
- Imposition by parents/relatives

1. **At present, how do you judge the choice made?**

- Positively
- Negatively
- I don’t know

1. **Year of course that you are attending: ______ (*If you are at 1^st^ year, go to question n. 23*)**
2. **In which range do you place the arithmetic average of grades of the exams you took?**

- 18-20.99
- 21-23.99
- 24-26.99
- 27-28.99
- ≥29

1. **Are your satisfied with your grade average?**

- Yes
- No, but it’s not a problem. Grade average is not my priority
- No, I want to work harder to improve
- No, but I am already working hard and I don’t think my grade average reflects my diligence

1. **Are you in time with the exams?**

- Yes
- No, but it’s not a problem
- No and it’s a problem

1. **Is there a psychological counselling in your university?**

- Yes
- No
- I don’t know

1. **Would you use it in case of need?**

- Yes
- No
- I don’t know

1. **How would you define the climate between your classmates?**

*(Choose only one answer that you think best suits you)*

- Friendly and relaxed
- Competitive and stimulating
- Competitive and hostile
- I don’t have an opinion yet

1. **Have you built friendships with a circle of classmates that are satisfying for you?**

- Yes
- Not yet, but I wish I will
- No, I don’t feel the need
- No, I don’t think that the friendships that I built are satisfying

1. **Do you have any worries about the future?** (*you can select* *more than one answer*)

- No, I think that the future is stimulating
- No, I am not thinking about my future at the moment
- Yes, I am worried about not measuring up to the profession
- Yes, I am worried about the choice of the specialty
- Yes, I am worried about the limited number of places for specialty/job

1. **Have you ever taken stimulants to improve your academic performance (exams, internships)?**

- No
- Yes. Which? (exclude coffee/energizing drinks) __________________

1. **Have you ever taken psychiatric medications during you university career?**

- No
- Yes. Complete the following table by writing the name of the medication(s) in the space with dots and placing a cross on the box indicating the respective frequency of use:

| Name of medication: | Rarely | Monthly | Weekly | Daily |
| --- | --- | --- | --- | --- |
| ………………….. |  |  |  |  |
| ………………….. |  |  |  |  |
| ………………….. |  |  |  |  |
| ………………….. |  |  |  |  |

1. **Are you seeing a psychologist/psychiatrist?**

- Yes
- No
